# Feasibility study of goal setting discussions between older adults and volunteers facilitated by an eHealth application: development of the Health TAPESTRY approach

**DOI:** 10.1186/s40814-018-0377-2

**Published:** 2018-12-12

**Authors:** Dena Javadi, Larkin Lamarche, Ernie Avilla, Raied Siddiqui, Jessica Gaber, Mehreen Bhamani, Doug Oliver, Laura Cleghorn, Dee Mangin, Lisa Dolovich

**Affiliations:** 1 McMaster University, DFM DBHSC, 5th Floor 100 Main St West, Hamilton, Ontario L8P 1H6 Canada; 2 McMaster University, DFM DBHSC, 3rd Floor, 100 Main St West, Hamilton, Ontario L8P 1H6 Canada

## Abstract

**Background:**

In keeping with the changing needs of the Canadian population, primary care systems need to become more person-focused in providing quality care to older adults. As part of Health TAPESTRY, a complex intervention to strengthen primary care for older adults, a goal setting exercise was developed and tested in an initial feasibility study, intended to foster collaboration between patients and providers.

**Methods:**

Participants—clinic clients—were recruited from the McMaster Family Health Team in Hamilton, Ontario. Five participants took part in the goal setting feasibility study phase I, which tested the functionality of a technology-enabled goal setting exercise between older adults and volunteers. Based on observations and feedback from volunteers, interprofessional team members, and older adults, the exercise was refined to include a guided survey and goals report. The goal setting survey is a list of probing questions designed based on SMART (specific, measurable, attainable, relevant, timely) goal setting strategies and goal attainment scaling (GAS). This was used in phase II, carried out with 16 participants, where the feasibility of goal setting and goal attainment with support from volunteers and interprofessional teams was tested. Volunteers carried out the goal setting survey via a tablet computer, a report of client goals was generated and sent to interprofessional teams, and client goals were discussed during clinic huddles. At 6 months of follow-up, clients self-evaluated their progress using GAS.

**Results and discussion:**

The goal setting exercise in phase I took an average of 24:45 (SD 11:42) minutes and yielded a diverse set of life and health goals. Goals identified by older adults were primarily focused on the *maintenance* of a certain level of activity or health state. Phase I work resulted in important changes to the goal setting process (e.g., asking about goal setting later in conversation, changing wording of questions) and development of a summary report of goals sent to the interprofessional team. In phase II, 44 goals were set by 16 participants during an average 7:23 (SD 4:26) minute discussion. Of these goals, 43.9% were characterized as health goals while 63.4% were characterized as life goals. Under the umbrella of Life goals, productivity featured most prominently at 22.9% of all goals. Goal attainment was not measured in phase I. In phase II, clients had an average weighted goal attainment score of 51.5. Considering client preferences for one goal over another, 68.8% of clients, on average, *at least partially achieved* the goals they had set.

**Conclusion:**

Goal setting as part of the Health TAPESTRY approach was feasible and provided interprofessional teams with client narratives that helped improve care management for older adults. The overall intervention—including the refined goal setting component—is being scaled and evaluated in a pragmatic randomized controlled trial.

## Background

### Person-focused care

The Alma Ata declaration of 1978 is a public health milestone that continues to serve as the guiding principle for primary health care interventions. According to the declaration, primary health care is “essential health care based on practical, scientifically sound and socially acceptable methods and technology made universally accessible to individuals and families in the community through their full participation and at a cost that the community and country can afford to maintain at every stage of their development in the spirit of self-reliance and self-determination” [1]. This encourages a person-focused paradigm that moves away from the disease-driven approach traditionally used and instead focuses on the whole person through health promotion at a holistic level with importance given to the combination of research evidence, patient priorities and preferences, and clinical state and circumstances [2–4]. Health systems around the world have increasingly tried to implement the various elements of the Alma Ata into their primary care strategies with varying degrees of success. In the USA, the Institute of Medicine, in their report *Crossing the Quality Chasm*, highlighted a holistic approach of reform involving all health care constituencies with six aims for improvement in making health care safe, effective, person-focused, timely, efficient, and equitable [5, 6]. Some of the rules applied to this system redesign included having care be customized according to patient needs and values, having the patient as the central locus of control, and shared decision-making with free-flowing information [5]. In Ontario, the *Living Longer, Living Well* report released in December 2012 to inform the Ontario Seniors Strategy called for a strengthening of primary care to support the needs of older adults with attention given to access to primary care providers, better coordination and communication between agencies such as Community Care Access Centres (CCACs) and primary care physicians, and additional resources for house calls [6].

pt?>Customization of care means that the system has the adaptive capacity to tailor care to individual patient choices and preferences. Empowering the patient to be the central locus of control involves providing patients with the information, support, and means to actively make decisions. Shared decision-making and free-flowing information means that patients should be able to easily access their own medical information and that communication between providers and patients should be effectively facilitated. In addition, a patient-centred approach has been proposed for those with multimorbidity [7]. The Royal College of General Practitioners in the UK, in their *Inquiry into Patient-centred Care in the 21st Century*, highlighted similar themes, defining patient-centred care as holistic- or “whole person”-flexible, tailored care, supported by collaborative relationships between providers and empowered patients [8]. In this paper, we use the term “person-focused,” as it goes beyond the facility visit and takes a whole person approach, encompassing the impact of personal narratives and contexts [9]. A key function of the primary health care system is to help people stay functionally independent (or “healthy”) recognizing that people are heterogeneous in terms of health and function, but everyone can have health-related goals. In Canada, Roy Romanow’s Report on the Future of Health Care in Canada highlights the fact that “the direction of our health care system must be shaped around health needs of individual patients, their families and communities,” focusing on ensuring that health providers are trained and supported in sharing expertise in an interprofessional team environment to perpetuate collaborative person-focused practice [10].

One of the central themes to all of these approaches for enhancing person-focused care is patient empowerment and tailored support for care planning. Agreement around goals of care between patients and providers demonstrates improved outcomes [11, 12]. Explicit processes that incorporate formal goal setting as part of care planning are a means of ensuring that care planning is person-focused and tailored to suit patient preferences and needs [13]. Supportive goal setting also provides patients with the resources, information, and connections to enhance self-management and improve health outcomes. Additionally, as another key tenet of improved primary care, collaboration and communication between patients and providers is enhanced by goal setting through the reinforcement of shared priorities [3]. However, studies on interdisciplinary approaches to providing care for community-dwelling frail older adults have been unable to demonstrate impact in part due to a lack of a standardized approach to goal setting as well as a lack of sufficiently responsive outcome measurement tools, especially in capturing progress on goals set in partnership with patients [14, 15].

### Goal attainment scaling

Individualized goal attainment scaling (GAS) systems are a means of evaluating the effectiveness of person-focused strategies that incorporate goal setting [3]. GAS has been validated in the literature and is a reliable tool in setting quantifiable person-focused goals and measuring the level of improvement towards achieving these goals. GAS was first developed by Kiresuk and Sherman (1968) in evaluation of mental health programs and has since been used in the evaluation of service delivery across many fields [16–18]. The strengths of GAS come in providing a means of measuring person-focused outcomes that are individualized yet can be scaled for comparison between and within groups. This is especially important in allowing for comparison of outcomes across a heterogeneous complex group such as older adult populations given multimorbidities and the effects of social determinants of health on this varied population [19].

GAS has demonstrated responsiveness in capturing change in frail older adults and is a valid tool to capture patient preferences, which could indicate judgments, around effectiveness of care as well as capturing clinically relevant change [19, 20]. It has also shown to be more responsive than other outcome measures alone in patients with complex disabilities, demonstrating an added value when used in combination with standard outcomes measurements to capture progress [21]. There is evidence for reliability, validity, and sensitivity of GAS [17, 19, 20, 22, 23]. Furthermore, empirical evidence supports the validity of goal setting for enhancing patient participation in care planning including in older adults with multiple chronic conditions [3, 24]. Studies have shown that older adults can successfully participate in goal setting, scaling, and assessment, making use of GAS feasible in this population [20, 24–26]. The GAS tool has been compared to other outcome measures such as the Canadian Occupational Performance Measure and found to be a sensitive albeit more time consuming tool than others; however, it demonstrates greater flexibility and better suits complex interventions where outcome measures can range beyond medical or rehabilitation goals [23].

It is important to note that GAS presents many challenges, and program-specific adjustments may be required to enhance feasibility. For example, GAS as originally described can be impractical for physicians to use for the following reasons: it is time consuming to engage in structured goal setting discussions with patients, including the time it takes to devise benchmarks for each of the levels to be assessed when only one level is ultimately used; use of numbers as labels for achievement can be discouraging to patients, especially negative and zero numbers; and the inability to score partial achievement can be a deterrent [18]. Other challenges include the comfort levels of providers with facilitating goal setting conversations, the nature of the goals set (i.e., too challenging or not challenging enough), and the subjectivity of benchmark levels [27]. In most applications of GAS, administrators have clinical training (in rehabilitation, long-term care, etc.); therefore, using non-clinically trained volunteers in the Health TAPESTRY model is also testing the utility of GAS applications by lay providers [28].

### The Health TAPESTRY approach

With the demographic shift towards increased life expectancy, it is important to consider the complexity introduced by living longer with chronic disease comorbidities [29]. Health systems need to develop the capacity to offer ongoing support to patients in addressing their holistic health care needs. It is with this motivation that Health TAPESTRY launched its team-based, technology-enabled, volunteer-supported approach to the provision of primary care services for this complex population of older adults [30]. This multi-component intervention creates times and space for interprofessional health teams to come together to discuss patient goals and needs based on health and lifestyle information collected by volunteers using a tablet-based application. As part of this overall approach, a goal setting exercise was developed to enhance person-focused care. A feasibility study was conducted to document the development of this goal setting exercise, its integration into the over-arching complex intervention, and its acceptability by the target audiences both at user and provider levels.

The objectives of Health TAPESTRY’s goal setting exercise include (1) providing volunteers with the tools to discuss and understand what matters to clients, (2) providing interprofessional health care teams with a better understanding of their patients and their needs, and (3) assessing Health TAPESTRY’s level of effectiveness in improving person-focused care and in helping clients achieve their goals. Health TAPESTRY’s goal setting component is rooted in the principles of person-focused care and the theory of planned behaviour, focusing on the need for “intention” as an important component of behaviour change [31]. This paper will describe the development and implementation of Health TAPESTRY’s goal setting process through a volunteer-administered web-based application (app). The findings from this effort are intended to strengthen the knowledge-base on the application of GAS and to provide further insight on the role of volunteers in serving as health liaisons between older adults and interprofessional health teams [32]. The resultant goal setting process will be incorporated into the Health TAPESTRY program. The overall program will be evaluated at a larger scale in future research with the hypothesis that by developing goals and associated treatment strategies—and by integrating this with other changes to the service delivery model—primary care will be more responsive and person-focused in meeting the health needs of older adults.

## Methods

### Design

**Fig. 1:**
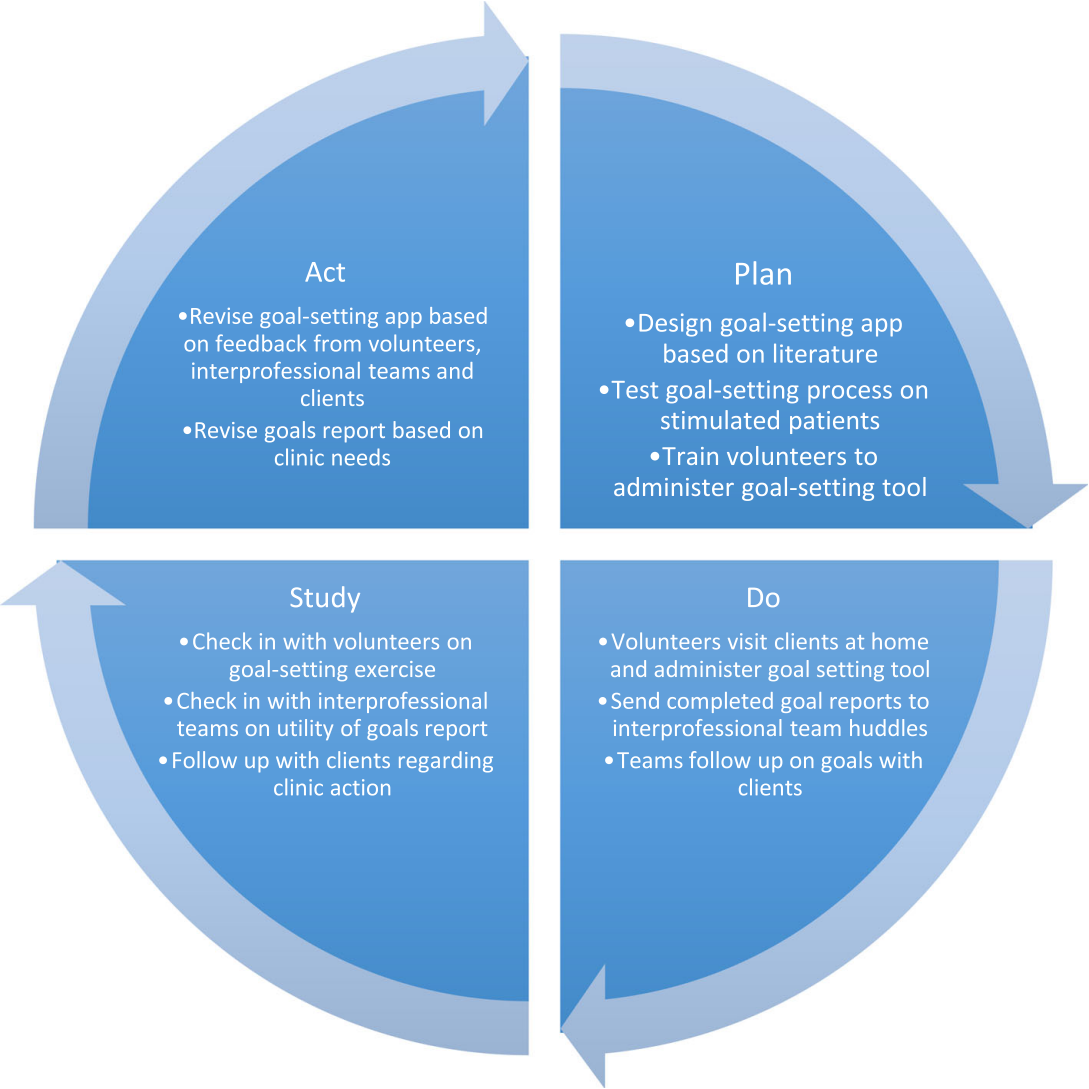
Plan-Do-Study-Act process for Health TAPESTRY’s goal setting pilot

This was a two-phase development and evaluation feasibility study. To test the goal setting exercise, two pilot phases were undertaken. Phase I of this pilot study tested the functionality of a technology-enabled goal setting exercise between older adults and volunteers. Volunteers were community members with training on how to engage with older adults and how to use a series of health surveys on a tablet computer to communicate basic health needs back to an interprofessional team at a primary care clinic. The objectives were to observe whether a goal setting conversation using a technology-enabled discussion guide or survey was practical, capture the experiences of volunteers and older adults taking part in the study, identify the types of goals older adults select, and inform improved iterations of the tool. Based on observations and feedback from volunteers and older adults, the exercise was refined. As a result, in phase II, important changes were made to the goal setting process (e.g., asking about goal setting later in conversation, changing wording of questions) and a summary report of goals was conceptualized and included in a report sent to interprofessional teams at the primary care clinics. Phase II was carried out with the objective of testing the feasibility of goal setting and goal attainment with support from volunteers and interprofessional teams. A Plan-Do-Study-Act (PDSA) model was applied in the development of the goal setting process in phase II [33]. This was done to ensure that the process was appropriate for all actors involved and that it was designed in a way that provided clients, volunteers, and interprofessional teams with a valuable tool for improved communication and linkages, see Fig. 1 for PDSA cycle and associated activities. Throughout the implementation of this exercise, volunteers and interprofessional team members were asked for their feedback on the goal setting process as well as the resulting goals and targets it was yielding. The project was approved by the Hamilton Integrated Research Ethics Board (#13-366).

### Participants and setting

Participants were recruited from the McMaster Family Health Team (MFHT) in Hamilton, Ontario. The MFHT consists of two sites. At the time of the study, there were approximately 32,000 patients, and 21 full-time equivalent family physicians, 74 family medicine residents, and 11 nurse practitioners, as well as registered practical nurses, social workers, occupational therapists, physiotherapists, pharmacists, system navigators, dietitians, physician’s assistants, a lactation consultant, and consulting specialists. In Health TAPESTRY, interprofessional teams include the nuclear interprofessional team (health care providers affiliated with MFHT), other health care providers (specialists), community organizations, or others identified by the nuclear health care team or client as being part of their care circle.

In phase I, a convenience sample of five participants aged 65 years or older were recruited from the MFHT. In phase II, 16 participants, different from the original five, were randomly selected from those participating in the initial phase of a randomized controlled trial (RCT) of Health TAPESTRY (ClinicalTrials.gov NCT02283723). The exclusion criteria included if the patient was deceased, had explicitly stated that they did not want to be part of a research project, resided in long-term care, or were receiving end of life care.

### Phase I: Initial experience with goal setting discussion during home visits

Clients were visited in their homes by a pair of trained community volunteers. Volunteer pairs included one undergraduate student and one experienced community member, usually a retired professional with more than 2 years of volunteer experience. Volunteers used a web app, accessed by the tablet’s internet browser, to navigate the conversation with clients. The app hosts multiple surveys and questionnaires as part of the overall Health TAPESTRY approach. Surveys administered by volunteers via the app included one on activities of daily living with questions focused on “What matters to you,” “Things that keep you healthy,” “What a good day looks like for you,” and “Description of self-management coping strategies.” The responses collected from the “What matters to you” questions informed subsequent iterations of the goal setting survey including identification of goal domains, example goals and goal areas.

### Phase II: Use of goal setting app to communicate client goals and measure goal attainment

#### Plan: Development of refined goal setting survey based on goals literature and volunteer interactions with standardized patients

**Fig. 2:**
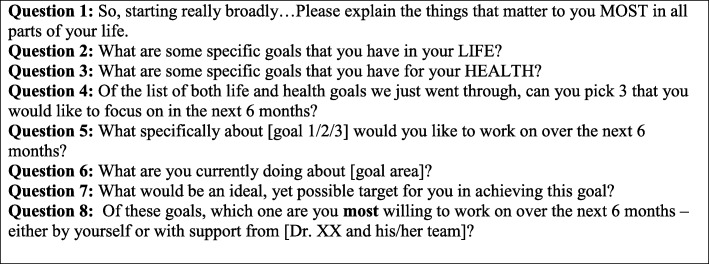
Goal setting questionnaire

Volunteers played a critical role in the development of the goal setting process including testing of the app with standardized patients. Four volunteer pair-standardized patient interactions were observed, recorded, and analyzed by the research team. A standardized patient is “a healthy person trained to portray the personal history, physical symptoms, emotional characteristics and everyday concerns of an actual patient,” in order to serve as a dynamic learning tool and to offer constructive feedback during training sessions. During volunteer pair- standardized patient interactions, clarity of the questions, ease of use, and appropriate pace of conversation were observed and evaluated. A follow-up discussion with volunteers and standardized patients assessed comfort level with the goal setting exercise, areas for improvement in phrasing of questions and natural flow of goal setting process. Based on this discussion, changes were made to the “What matters to you” survey and the app (see Fig. 2 for the survey questions). SMART goal setting principles (setting specific, measurable, attainable, relevant and timely goals) were applied to the revised survey development [34]. In addition, principles of GAS were applied to ensure that goals were measureable (i.e., change in level of goal attainment from baseline) and to ensure that perceived (subjective) importance of specific goals by the client was incorporated into the measurement through weighting these goals. The app provides the option of prioritizing goals at the onset and then allows clients to self-identify their level of goal attainment during follow-up.

Moreover, volunteers provided feedback on client home visits, which was used to inform goal categories. As a result, the app includes a sample bank of life and health goals to help volunteers guide discussion should the client feel stuck in identifying goals.

#### Volunteer training

An in-person training session was held for all volunteers that consisted of three parts: (1) introduction of the goal setting survey with some background provided on the philosophy of goal setting and behaviour change motivation, (2) small group goal setting discussions with actors hired to act as standardized patients, and (3) feedback from standardized patients on strengths and weaknesses of the discussions.

An adapted Objective Structured Clinical Examination (OSCE) format was used to evaluate the readiness of volunteers and comfort level with the goal setting process. In small groups, volunteers were exposed to 15-min simulated home encounters with standardized patients (who portrayed a representative sample of potential encounters with older adults). Feedback from this exercise suggested that volunteers were much more comfortable having the goal setting discussion after having practiced with standardized patients.

### Do: Goal setting in the client’s home

At the start of the visit to the client’s home, volunteers used a series of Health TAPESTRY questions about their daily habits and activities to make a connection and start the conversation. Volunteers then used the goal setting app to go through the four steps on the goal setting survey: (1) identifying goal areas, (2) setting SMART goals, (3) setting specific goal targets to be measured with GAS, and finally (4) closing the conversation. Volunteers encouraged clients to set at least three goals, but if clients could not come up with this many, the visit continued as usual. Three to five goals were identified as a feasible number to provide a reasonable snapshot of client priorities [21].

#### Sharing client goals

**Fig. 3:**
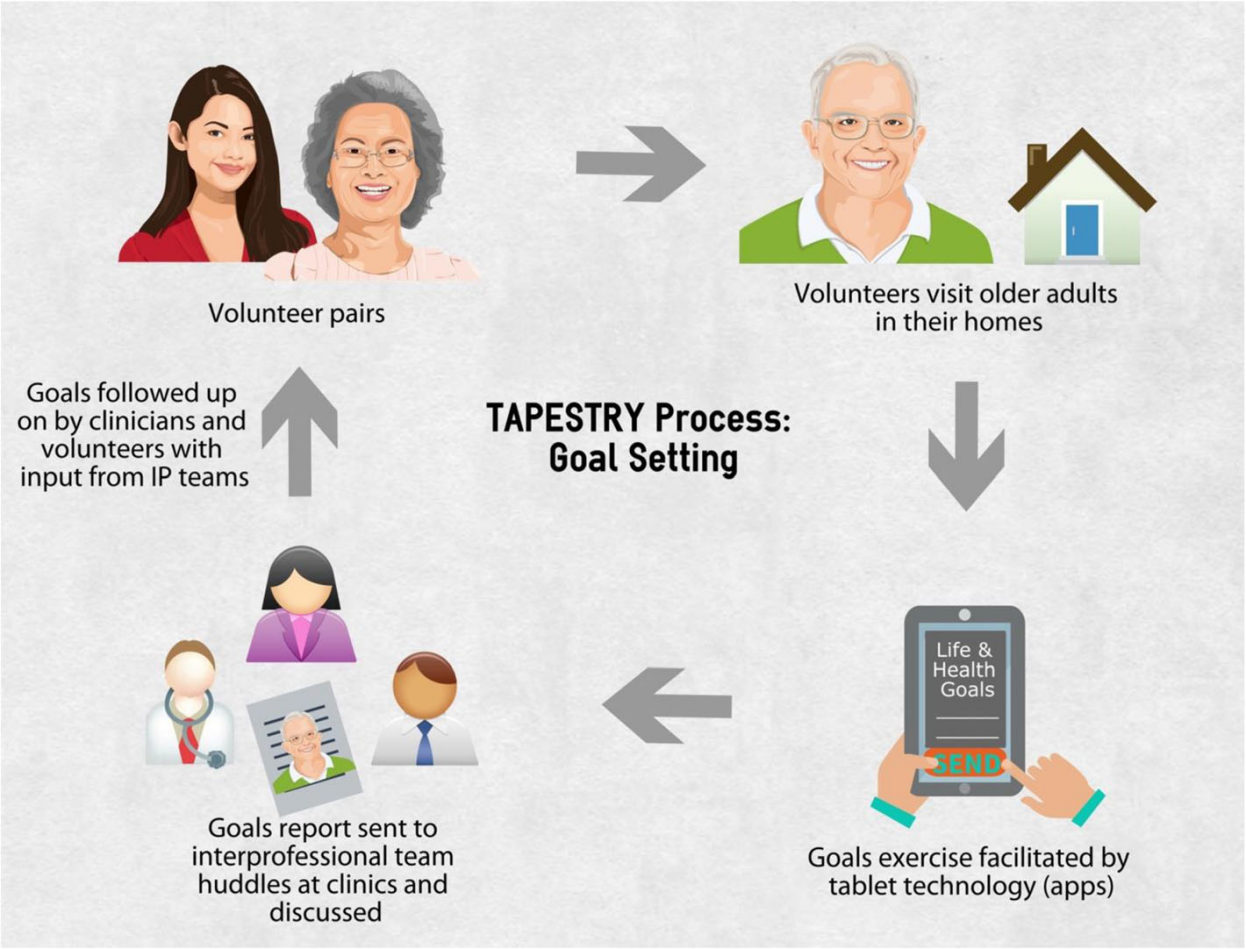
Health TAPESTRY goal setting process

Client goals and goal targets were recorded on the app and became part of the summary report of volunteer visits. This report included the three goals set and highlighted the one prioritized by the client. No client set more than three goals and all goal prioritization was done at the onset of the study. The report was sent to the family physician and to an in-clinic “huddle” team of allied health professionals who triaged reports from their own interprofessional lenses. The health care providers then decided on a course of follow-up based on both the client’s goals and results from the other health and social wellbeing surveys conducted by volunteers during their visit, see Fig. 3 for the overall goal setting process. This approach was meant to be complementary to the existing clinic approach of managing patients, giving health care providers another piece of the puzzle to better understand what their patients may need without investing a lot of time in having a full goals-focused conversation themselves. They can instead follow up with clients from where the report leaves off. Health TAPESTRY research team members were present during interprofessional team meetings in order to capture the process of project implementation accurately and apply this to future design of the program. Elements captured include the types of health or social providers mobilized to address client goals, follow-up plans for clients and resulting clinic visits by clients. Challenges voiced by the interprofessional teams in addressing client goals were also captured.

#### Follow-up

To maintain the relationship between clients and volunteers, as well as to sustain a link to the interprofessional team, Health TAPESTRY volunteers returned to clients’ homes 3 months after the initial goals survey to check in with clients regarding their progress on the goals they had set. The 3-month follow-up survey was designed to capture any enablers and barriers to goal attainment by clients. Volunteers reminded clients of their goals and then asked how things were going. If clients were having trouble achieving progress, volunteers encouraged them to share any barriers they may have been facing as well as any resources they had at their disposal to support goal attainment. These barriers and enablers were shared with the interprofessional team on a brief follow-up report sent to the clinic huddles. Six months after the initial goals survey, clients were visited by volunteers once again and asked about progress towards their goals as well as any new barriers and enablers. Clients were asked to provide a number on a five-point scale to express their perceived level of goal attainment.

#### Data analysis

Interviews with older adults and volunteers were transcribed and analyzed thematically, looking at challenges and successes in carrying out the goal setting exercise. Clinic huddle observations for interprofessional team course of action for each client were captured by a Health TAPESTRY team member and entered into Excel, exploring what goals were highlighted, what visits were scheduled, and what other follow-ups were made. Key challenges articulated by interprofessional teams were also captured by the observer. Goal attainment was measured using data captured by volunteers on the goal setting app. Barriers and facilitators to goal attainment were also captured on the goal setting app and thematically grouped. Data were analyzed using descriptive statistics and GAS scores. Types of goal areas and their dominance were identified as well as common barriers and facilitators mentioned by clients. Feedback from clients and volunteers and the resulting changes made to the app for phase II have also been highlighted. GAS scores were identified using client responses and perceptions of progress at the 6-month mark. Due to evidence in the literature on the lack of flexibility in GAS to measure partial achievement, the team decided to allow for partial achievement to rank as “0” on the scale [18]. Therefore, based on client responses, researchers assigned GAS scores of − 2 (worse than before), − 1 (same as before), 0 (partial achievement), + 1 (expected achievement), and + 2 (exceeded expectations) in reference to the goal targets previously set by clients. Overall GAS scores were calculated using the following formula where *w*_i_ is the weight assigned to the *i*th goal and *x*_i_ is the numerical value achieved between − 2 and + 2.

$$ \mathrm{Overall}\kern0.5em \mathrm{GAS}=50+\frac{10\sum \left({w}_i\ {x}_i\right)}{\sqrt{0.7\sum {w_i}^2+0.3\sum {w_i}^2}} $$

## Results and discussion

### Study: Phase I feasibility study

Table 1 shows the sample characteristics for phase I. The first question of “What matters to you in your life?” was used to start the conversation and facilitate the rest of the discussion. The questions on specific life and health goals resulted in primarily goals around *maintaining* some level of activity or health state, which is in line with the literature around a preference for maintenance goals for older adults [35, 36]. Table 2 highlights the types of responses to questions on life and health goals. These, together with reflections from the literature, were used to determine the eight goal domains used to provide volunteers with thematically organized goal samples. See Table 3 for the goal domains as well as examples of goals under these domains.

**Table 1:** Sample characteristics for phases I and II pilot

Variable	Phase I pilot	Phase II pilot
Sample size	5	16
Age, mean (SD)	76.5 (9.1) years	80.0 (6.1) years
Gender (*n*, % women)	7 (87.5%)	8 (50%)
Length of discussion, mean SD)	24:45 (11:42) min	7:23 (4:26) min

**Table 2:** Responses to questions on life and health goals

Health goals	Life goals
Maintain current level of mobility	See children more often
Increase rate of exercise	Travel
Better manage pain post-surgery

**Table 3:** Goal domains and example of goals under each domain

Health goal domains	Examples	Life goal domains	Examples
Diet	Manage weight Manage salt intake	Productivity	Do more volunteer work Pick up a new hobby
Physical activity	More frequent exercise Improve flexibility	Social connection	Join a club See family more often
Rehabilitation	Manage pain Decrease rigidity	Other (finances, faith)	Travel more Manage finances
Smoking/alcohol	Smoke less often Drink fewer drinks		
Medical	Manage blood pressure Manage medications		
Mental health	Manage stress Take on relaxation activity		
Maintain health	Remain at same level of exercise Continue to exercise the mind		

### Study: Phase II feasibility study

**Fig. 4:**
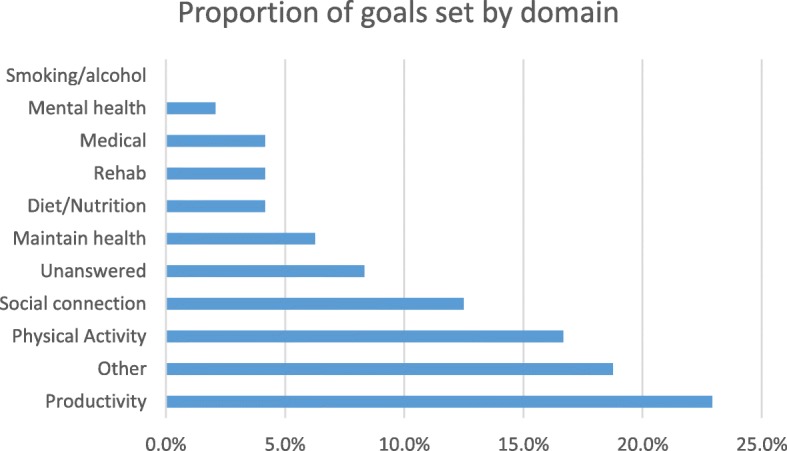
Number of goals set per domain

Table 1 shows the sample characteristics for phase II. Of 16 clients, 13 set three goals each. Clients who did not set all three goals set at least one. Of the 44 goals set by 16 clients, productivity goals (for example, volunteering, hobbies) were the most prominent at 22.9%, followed by goals defined as “other” at 18.8%, which included goals around financial management, travel, sleep, and driving ability. Physical activity and social connection goals represented 16.7% and 12.5% of goals set, respectively (see Fig. 4 for number of goals set by goal domain). In total, 43.9% (*n* = 18) of goals set were characterized as “health goals” while 63.4% (*n* = 26) of goals set were characterized as “life goals.”

By having volunteers facilitating clients’ story-telling to their health care providers, Health TAPESTRY ensured that client narratives became part of a holistic picture that interprofessional teams could reference and use to provide tailored person-focused care. This encouraged interprofessional teams to involve the appropriate types of providers to address the needs of particular clients. For example, knowledge of housing-related challenges that were affecting client health led to involvement of system navigators and mobilization of resources to support clients in improving their situations. However, the process is not without challenges. Volunteers communicated that the reasons more than one goal was not set in some cases were due to the client’s inability to think of specific goals, client frustration with repetition during the goal setting exercise, and problems with the technology, meaning that goals were not retained on the app. This feedback, together with other general comments from volunteers, allowed the Health TAPESTRY team to refine the technology to be more user-friendly as well as to reorganize the survey to be less repetitive for the randomized controlled trial.

Three-month follow-up reports, focused on barriers and enablers, demonstrated the heterogeneity of older adults and the external factors that affect their quality of life. While some of the barriers—and even some of the goals set—were not amenable to intervention by health care providers, having this narrative from their patients provided valuable context, such as an indication of their patients’ lived environments and strength of social support networks. This is especially important in the setting in which we carried out our study as the clinics are teaching practices, which means that patients see more than one provider regularly and therefore have less continuity with their provider. The intention of this approach was to illustrate patients’ social determinants of health, perceived barriers and enablers, desires, and unattended medical conditions impeding high quality of life. Of 16 clients, five cited no barriers and 11 cited a barrier for at least one of their set goals. Common barriers reported by clients are shown in Table 4.

**Table 4:** Example goals and associated barriers reported by participants

Example goal	Barrier
Example 1. Goal: to walk as exercise.	“My legs do not allow me to walk. I develop pain whenever I walk.”
Example 2. Goal: to socialize more	“I just do not feel like going out.”
Example 3. Goal: monitor and maintain diet set by dietician.	“Chocolate chip cookies”
Example 4. Goal: getting more involved with community initiatives	“did not have enough time to get into more initiatives- knew brother was going to be getting sick so I knew I would need time to help him”
Example 5. Goal: to keep dancing	“Can’t put weight on the ankle, can’t step with ball of foot first”

While some of these barriers highlight specific medical issues that health care providers may or may not know about (examples 1 and 5, Table 4), others highlight warning signs for other potential challenges to optimal aging. For instance, example 4 could alert providers to a potential for development of caregiver burden, while example 2 may be displaying early signs of withdrawal or potential depression. These clues to the clients’ context strengthen the role of primary care in disease prevention and health promotion.

At 6-month follow-up, client perceptions of their level of goal attainment were captured. GAS was used to provide an attainment score. When setting goals, clients were asked which goal they were most willing to work on; in the weighted GAS measurements, this goal has been weighted twice as much as other goals set. Both weighted and unweighted GAS scores are provided.

The average weighted GAS score was 51.5, and average unweighted GAS score was 53.2. When weighting scores, six of 16 clients had GAS scores of above 50, which signify that they had mostly met or exceeded expectations on their goals. Five had mostly stayed at their baseline levels or regressed on their goal targets signified by cumulative GAS scores below 50. And five clients had, on average, partially achieved goals. With no weight, eight had GAS scores of above 50, four had scores below 50 and the four remaining had scores of 50. Therefore, taking into account client preferences for one goal over another, 68.8% (*n* = 16) of clients on average at least partially achieved the goals they had set, see Table 5 for a summary of GAS scores.

**Table 5:** GAS scores

Client	Goal 1 scale	Goal 2 scale	Goal 3 scale	GAS score unweighted	Weighted goal	GAS score weighted
1	0	2	−1	55.8	Goal 3	50.0
2	2	1	2	78.9	Goal 1	78.6
3	0	0	2	61.5	Goal 1	58.2
4	1	0	−2	44.2	Goal 2	45.9
5	2	0	−1	55.8	Goal 3	50.0
6	1	1		61.5	Goal 3	58.2
7	1	1	−2	50.0	Goal 3	41.83
8	1	−1	NA	50.0	Goal 3	50.0
9	−1	− 1	2	50.0	None	50.0
10	1	2	0	67.3	Goal 1	45.9
11	−1	1		50.0	Goal 3	50.0
12	1	−2	−1	38.5	Goal 1	45.9
13	NA	1	NA	55.8	Goal 3	54.1
14	0	1	1	61.5	Goal 1	54.1
15	−1	−1	−1	32.7	Goal 1	58.2
16	−1	− 1	NA	38.5	Goal 2	33.7
Average GAS scores (*n* = 16)				53.2 ± 11.7		51.5 ± 9.6

Note. NA = Not applicable

### Adapting the survey based on data from phases I and II

Throughout the implementation of this exercise, volunteers and interprofessional team members were asked for their feedback on the goal setting process as well as the resulting goals and targets it was yielding. Based on volunteer feedback, the placement of the goal setting exercise was moved from the beginning of volunteer home visits to the end. Volunteers noted that once they had gone through other Health TAPESTRY surveys on the app, the client was more comfortable and prepared to share his/her life and health goals with them. This finding is similar to those of other studies where feasibility and success of goal setting were dependent on willingness by both patients and providers to discuss goals and to devise treatment plans, as well as building trust and rapport prior to engaging in goal setting discussions as patients found these too personal in nature to discuss during first visits [24]. The change in placement was therefore applied across all volunteer visits and the goals survey was moved to the end of volunteer visits, resulting in more robust goals being set by clients.

The adoption of the Health TAPESTRY approach by interprofessional teams was a key element to the success of the goal setting exercise and its outputs. Interprofessional teams adopted the Health TAPESTRY approach in three phases: (1) discussion/development, (2) initial implementation/pilot, and (3) ongoing maintenance. Health TAPESTRY representatives were present at clinic huddles held by the interprofessional team; this is how the clinical team’s recommendations, concerns, and interpretations of goals were relayed back to the research team and taken into account in the redesign of the goals app and relevant components of the report. Feedback from interprofessional team members provided the research team with insight into the kind of information that would help the team best understand clients’ goals and needs. They also asked for goals to be placed at the top of the report in order to set the context from the start through the clients’ narratives. This feedback changed the survey responses that were being featured on client reports, resulting in more coherent goal summaries outlined on client reports and a generally better interpretation of these goals by the interprofessional team during clinic huddles.

## Discussion

Goal setting using structured GAS processes as part of the Health TAPESTRY approach provided the interprofessional team with client narratives that helped identify goal priorities and barriers and facilitators to achieving a client’s life and health goals. By highlighting self-identified goals, this approach provided important background information to interprofessional teams and facilitated person-centred care. Older adults tended to set goals across a wide range of goal areas, but often focused on maintenance versus improvement; it is important for providers and others facilitating goal setting conversations to appreciate the value of the maintenance of current states in this target population.

This two-phased feasibility study, as an iterative PDSA cycle that included engagement with key actors (clients, volunteers, and interprofessional team members), identified multiple improvements to the process of goal setting, leading to the majority of participants at least partially achieving the goals they had set after 6 months. For example, goal setting conversations were initially held as the first conversation during volunteer visits but were changed to a later conversation to allow clients time to develop stronger trust and a feeling of comfort and security in the volunteers and the program. As seen in other GAS interventions, goal setting was perceived by clients as a personal conversation and not part of their medical treatment [24]; therefore, more trust-building by volunteers was required before clients were better able to discuss life and health goals. No pilot clients refused to have the goal setting conversation; however, various challenges remain in making this conversation as comfortable and effective as possible. Using volunteers to facilitate the conversation in clients’ homes likely made it easier for clients to discuss what mattered to them as it limited some of the deference to medical opinion seen in clinic settings [24]. However, effective transfer of the information from the client-volunteer interaction to the healthcare team was essential to allow the team to continue engagement with the client on health goals and priorities. The feasibility study allowed for continuous improvement of the goal setting exercise, resulting in more effective integration of this piece into the multi-component Health TAPESTRY initiative at scale.

The goal setting process is now taking place within Health TAPESTRY to connect people living in their homes with their interprofessional primary care team and facilitate discussions on person-tailored treatment plans. The weekly team huddles and conversations around goals have been integrated into the clinic’s practice demonstrating a sustained and feasible process. Understanding clients’ goals and social determinants of health provides clinicians with an improved plan of how to manage care and enhances communication and cooperation between clinicians and clients [37]. A similar study found goals of older adults to fall primarily under leisure activity, self-care, and productivity [3]. However, in practice, the challenges in goal setting make it difficult to achieve buy-in from both groups. For example, in a study of older patients having goal setting conversation with clinicians for shared decision-making, participants felt goal setting was a good idea but not possible in practice due to the time requirements and the effort needed to set specific goals that are not generalizable across all patients [24]. The app used in this study was designed to enhance efficiency by more smoothly guiding the goal setting conversation.

A recent systematic review of tools for clinicians to record overall patient preferences and priorities for care [38] identified only one tool that has been developed and tested and that is suitable for eliciting preferences of older persons with multimorbidity [39]. In Fried et al., 81 participants were asked to rank four outcomes (maintaining independence, staying alive, reducing/eliminating pain, and reducing/eliminating other symptoms) on a visual analogue scale as either most or least important [39]. The outcome set was pre-defined and so limited to a narrow but important set of outcomes; however, our study found that older adults tended to set goals across a wide range of goal areas highlighting the need for processes that can personalize the goal and priority setting processes for use within clinical encounters as well as for research purposes.

When using technology to facilitate goal setting conversations, the tools must be sufficiently user-friendly and functional so as to encourage maximum engagement in the exercise. A systematic review of internet-based tools to support lifestyle modification found that adherence to the tool and follow-up were primary challenges and a “soft touch” component was required to increase engagement and effectiveness [40]. “Soft touch” involved peer support and regular check-ins and monitoring to allow patients to discuss their challenges [40]. In Health TAPESTRY, the soft touch was provided through the relationship with volunteers.

Regular client follow-up about their goals revealed important barriers that enabled interprofessional health teams to address and support client goal attainment. These barriers can often be non-health related, such as social isolation, making it necessary for health teams to engage with other community services in order to sufficiently address patient needs. Studies have suggested that despite having goal setting discussions with patients, providers are limited by their professional roles and available resources in working with patients and may therefore privilege certain goals, resulting in the finalized goals being similar to standard guidelines for treatment rather than truly reflective of patient needs or preferences [41]. It therefore becomes important to ensure that the system within which goal setting is being integrated allows providers to go beyond traditional treatment limitations and provide more holistic plans for their patients [41].

Effectiveness and efficacy measures in provision of holistic, person-focused care are difficult to capture as evidenced by a systematic review [42]. Criticism of GAS for measuring outcomes is that it may be too sensitive and lead to capture of changes with little clinical importance [20]. A key objective of Health TAPESTRY is to provide person-focused quality care that enhances the client’s sense of self-efficacy in optimal aging; therefore, an outcome measure that prioritizes the client’s attainment of personal goals, even where clinical importance may be less significant, is an appropriate measure for this study. It has been noted that goal-oriented care may contradict more disease-based performance measures for clinicians, which creates contrasting definitions of good quality care [43]. However, Health TAPESTRY is part of an effort to shift the care paradigm towards person-focused care which requires prioritization of clients’ preferred outcomes over disease-based outcomes. Other outcome measures have been used to assess the Health TAPESTRY intervention as a whole and will be described as part of the broader RCT studying the complex intervention. The findings from this pilot study will be applied to future scale-up evaluations of the Health TAPESTRY approach to ensure that client goals are being captured, communicated, and acted upon through the support of volunteers and interprofessional teams.

### Challenges

While piloting the goals discussion, many limitations and challenges arose. The Health TAPESTRY team deviated from the standard approach to GAS of having clients set goals and targets with a health care provider and instead chose to conduct the goal setting conversation between clients and volunteers. This was done to alleviate the demand on the time of overburdened health care providers and instead facilitate the process of gaining an informed understanding of what matters to clients in the context in which they live, without increasing provider workload. However, this also meant that some of the goals set were uninformed by medical knowledge in terms of their feasibility; therefore, some clients set goals and were told that there was not much that could be done for them by their providers. To address this, the second wave of volunteers were trained to pre-empt the goal setting conversation by highlighting that it is a first step and clients may be refining their goals with their interprofessional teams.

In a similar vein, clients were more likely to set life goals, which resulted in goals that were beyond the scope of the interprofessional teams. This raised ethical questions for the research team as the study had unveiled social challenges faced by clients, but the interprofessional team did not have the mechanisms to deal with these issues. To tackle this, Health TAPESTRY has been working on strengthening community engagement to ensure that socioeconomic alerts raised by volunteers can be addressed by the appropriate professionals.

With the breadth of information provided on client reports, it is a challenge for interprofessional teams to address every item. For example, during clinic huddles, while goals were discussed for every client in the pilot, recommendations from interprofessional teams for client-specific follow-up were made only in reference to clients’ goals for 4 out of 16 pilot participants. However, where clients were brought in for clinic appointments, they usually had a discussion around their goals with their providers. It is worth noting that a recent RCT on articulating quality of life goals prior to physician encounters revealed that the process reduced physician empathy [44]. Therefore, future iterations of the goal setting exercise should pay special attention to its impact on how the process affects client-physician interactions.

Another challenge was the study population itself. Clients included older adults who were not frail but wanted to continue living well and some who felt they were too old for goal setting. The conversation therefore required much more thorough reflection and time to identify specific and meaningful goals. It is possible that the time we allotted via our volunteers was insufficient in capturing the most relevant goals for this population. Over time, with strengthened relationships with volunteers, this can be overcome.

## Conclusion

The goal setting component of the Health TAPESTRY intervention is intended to provide an opportunity for clients and volunteers to build rapport, for clients to share their stories and to highlight what matters to them in optimizing their healthy aging, and for the interprofessional team to better understand the context in which their clients are aging as well as the external influences that may be affecting clients’ self-perceived quality of life. The extended narrative that the goal setting discussion provides also allows interprofessional teams to reflect on their individual and collective roles when caring for clients and to tailor care to better meet client needs. Goal setting is one piece of the Health TAPESTRY puzzle, and it is an important one in ensuring that the Health TAPESTRY approach is person-focused and provides a clear avenue for the client’s voice to be better heard. Health TAPESTRY’s development has been and will continue to be an iterative process that learns from the feedback loops it creates and remains cognizant of the changing factors that could be affecting its effectiveness. This is done by establishing clear communication mechanisms for feedback and re-evaluation between volunteers, interprofessional teams, clients, and the Health TAPESTRY team, as evidenced by the adjustments made to the goal setting exercise throughout its implementation while retaining fidelity to its primary goal of promoting person-focused care.
